# Stroke-derived neutrophils demonstrate higher formation potential and impaired resolution of CD66b + driven neutrophil extracellular traps

**DOI:** 10.1186/s12883-022-02707-0

**Published:** 2022-05-20

**Authors:** Angeliki Datsi, Laura Piotrowski, Markella Markou, Thomas Köster, Isabelle Kohtz, Kerstin Lang, Sabine Plöttner, Heiko Udo Käfferlein, Burkhard Pleger, Ramon Martinez, Bogdan Pintea, Roland Fried, Marcus Müller, Rene Chapot, Konstantinos Gousias

**Affiliations:** 1 Institute for Transplantation Diagnostics and Cell Therapeutics, Heinrich-Heine-University Düsseldorf, 40225 Düsseldorf, Germany; 2 Medical School, Rheinische Friedrich-Wilhelms University of Bonn, Sigmund Freud Strasse 25, 53121 Bonn, Germany; 3 Department of Neurology and Psychotraumatology, BG Klinikum Duisburg, Großenbaumer Allee 250, 47249 Duisburg, Germany; 4 Department for Diagnostic and Interventional Radiology, University Hospital Bonn Venusberg-Campus 1, 53127 Bonn, Germany; 5 Ruhr University Bochum, Universitätsstraße 150, Bergmannsheil Bochum, 44801 Bochum, Germany; 6 Institute for Prevention and Occupational Medicine (IPA) Ruhr University Bochum (IPA), Bochum, Germany; 7 Department of Neurology, University Hospital Bergmannsheil Bochum, Bürkle-de-la Camp Platz 1, 44079 Bochum, Germany; 8 Department of BG Neurosurgery and Spinal Surgery, University Hospital Bergmannsheil Bochum, Bürkle-de-la Camp Platz 1, 44079 Bochum, Germany; 9 Statistics in the Biosciences, TU Dortmund University, Vogelpothsweg 87, 44221 Dortmund, Germany; 10 Department of Neurology, St Marien Academic Hospital Hamm, St Paulus Corporation, Knappenstrasse 19, 59071 Hamm, Germany; 11 Department of Radiology and Neuroradiology, Alfried-Krupp-Hospital Rüttenscheid, 45131 Essen, Germany; 12 Department of Neurosurgery, KLW St Paulus Corporation, St Marien Academic Hospital Lünen, Westfälische Wilhelms-University Münster, Altstadtstrasse 23, 44534 Lünen, Germany; 13 Medical School, University of Münster, Domagkstrasse 3, 48149 Münster, Germany; 14 Medical School, University of Nicosia, Ilia Papakyriakou 21, 2414 Nicosia, Cyprus

**Keywords:** Stroke, Thrombus, Neutrophil-Extracellular-Traps, CD66b +, Immunothrombosis

## Abstract

**Background:**

Recent evidence suggests a merging role of immunothrombosis in the formation of arterial thrombosis. Our study aims to investigate its relevance in stroke patients.

**Methods:**

We compared the peripheral immunological profile of stroke patients vs. healthy controls. Serum samples were functionally analyzed for their formation and clearance of Neutrophil-Extracellular-Traps. The composition of retrieved thrombi has been immunologically analyzed.

**Results:**

Peripheral blood of stroke patients showed significantly elevated levels of DNAse-I (*p* < 0.001), LDG (*p* = 0.003), CD4 (*p* = 0.005) as well as the pro-inflammatory cytokines IL-17 (*p* < 0.001), INF-γ (*p* < 0.001) and IL-22 (*p* < 0.001) compared to controls, reflecting a T_H_1/T_H_17 response. Increased counts of DNAse-I in sera (*p* = 0.045) and Neutrophil-Extracellular-Traps in thrombi (*p* = 0.032) have been observed in patients with onset time of symptoms longer than 4,5 h. Lower values of CD66b in thrombi were independently associated with greater improvement of NIHSS after mechanical thrombectomy (*p* = 0.045). Stroke-derived neutrophils show higher potential for Neutrophil-Extracellular-Traps formation after stimulation and worse resolution under DNAse-I treatment compared to neutrophils derived from healthy individuals.

**Conclusions:**

Our data provide new insight in the role of activated neutrophils and Neutrophil-Extracellular-Traps in ischemic stroke. Future larger studies are warranted to further investigate the role of immunothrombosis in the cascades of stroke.

**Trial registration:**

DRKS, DRKS00013278, Registered 15 November 2017, https://www.drks.de/drks_web/navigate.do?navigationId=trial.HTML&TRIAL_ID=DRKS00013278

**Supplementary Information:**

The online version contains supplementary material available at 10.1186/s12883-022-02707-0.

## Background

Mechanical thrombectomy (MT) combined with intravenous thrombolysis (IVT) appears as the superior therapeutic choice for recanalization after large artery strokes [1]. However, despite impressive breakthrough achievements in endovascular retrieval procedures no similar advances have been noticed in the pharmacological therapy since its establishment in the ‘90s [2]. The use of recombinant tissue plasminogen activator (rtPA) demonstrates strong limitations [3, 4]; even if applied within the first hours of symptoms’ onset only a small fraction of patients with large artery occlusions may benefit from arterial reperfusion [5]. The clinical failure of rtPA-IVT may rely on our limited knowledge about the underlying pathomechanisms of thrombogenesis. Analysis of the cellular thrombus composition as well as of potential circulating thrombogenic factors in serum of stroke patients may improve our understanding.

Recently, neuroinflammation has emerged as a novel key regulator of arterial thrombosis in stroke patients [6–14]. In the context of the so called immunothrombosis neutrophil markers, such as CD66b may be activated or neutrophil extracellular traps (NET) may be released, potentially contributing to the rtPA failure. Since NET comprise a network of extracellular chromatin fibers, they may form scaffolds which enclose platelets and subsequently activate the intrinsic coagulation cascades promoting thrombosis [6].

Naturally, the process of NET formation is an important regulator of innate immunity and mainly initiated upon contact with microbes by releasing antimicrobial traps composed of chromatin and neutrophil proteins from their nucleus or mitochondria. The formation of NETs can be initiated by ROS, the inflammasome or other triggers, leading to chromatin decondensation, associated with antimicrobial proteins. Upon ROS production the neutrophil elastase (NE) translocates to the nucleus, where it cleaves the histones. There it comes to chromatin decondensation, which is enhanced by the metalloprotease MPO [15–17]. Noteworthy, NETosis is an important player of immunothrombosis and potential therapeutic target also in Covid-19 [18].

Our study aims to analyze the role of immunothrombosis in generating large intracranial artery occlusions. For this scope we compared the immunological profile of stroke patients and controls. Additionally, we studied the composition of retrieved thrombi. Further in vitro stimulation of patients’ and controls’ neutrophils aimed to reveal their different thrombogenic potential.

## Methods

### Patients

We analyzed prospectively 48 (consecutive adult (>18 years) patients with acute ischemic stroke as a consequence of a large unilateral arterial occlusion, i.e. occlusion of intracranial arteria carotis interna (ACI), arteria cerebri media (ACM) or arteria basilaris/vertrebralis, who underwent mechanical thrombectomy (MT) at the Neuroradiological Department of Alfried Krupp Hospital in Essen between February and May 2018. 16 age matched patients with low back pain treated at the Neurosurgical Departments of St Marien Academic Hospital of Luenen and of BG Bergmannsheil University Hospital of Bochum served as control group. Patients suffering from an active cancer disease under chemotherapy or from an active bacterial or viral infection, with a history of autoimmune disease or under immunomodulatory medication were excluded from our study. Patient charts were reviewed and all pertinent clinical data were entered into a computer-based database. Patients for whom critical parameters, such as onset time of stroke symptoms, could not be clearly determined were excluded. This left 35 stroke patients and 16 controls for the final analyses. The study was approved by the Ethic Committees of the Ruhr University of Bochum (ethics committee vote 17–6060) and of the Westfaelische Wilhelms University of Münster (ethics committee vote 2020–310-f-S) (German Clinical Trials Register-ID: DRKS00013278). All methods were carried out in accordance with relevant guidelines and regulations. Inform consent was obtained from all patients or legal representatives.

MT was performed as described previously [4]. The time of IVT and the conservative treatment in general were in line with the German guidelines for acute therapy of ischemic stroke-recanalization therapy [4, 5]. Peripheral blood was collected prior to MT and serum as well as peripheral blood mononuclear cells (PBMCs) were obtained upon density gradient centrifugation performed within 4 h after withdrawal (median time 2 h).

The demographic and clinical categorical variables studied were sex (m/f), National Institutes of Health Stroke Scale (NIHSS) at admission, after MT and at discharge, onset time of stroke symptoms (< 4.5 vs. ≥ 4.5 h), comorbidities, Charlson Comorbidity Index (CCI) in general (0 vs. 1–2 vs. >2) or each comorbidity in particular (yes/no, Table 1), location and site of arterial occlusion, history of older demarcated infarct (yes/no) or atrial fibrillation (AF) (yes/no), treatment modalities (MT vs. MT and IVT), thrombolysis in cerebral infarction (TICI) score of recanalization, intake of novel oral anticoagulants, vitamin K antagonists, or antiplatelet drugs. Age and the expression values of our laboratory parameters were studied as continuous variables. In case of serum and PBMC, this included absolute counts of WBC, neutrophils, DNAse-I, low-density granulocytes (LDG), CD4 cells as well as intracellular IL-17, IL-22 and INF-γ production by CD4 cells. Furthermore, we analyzed the relative frequencies and absolute counts of isolated neutrophils, in particular of CD66b cells. In case of thrombi, the expression of CD66b and presence of NETs was assessed.

**Table 1:** Study group demographics

Variable		stroke patients (*n* = 35)	controls (*n* = 16)	*p* value
Males		18 (51.4%)	7 (43.8%)	n.s.
Age median (median, range)		77 (70.9, 26–98)	76.5 (70.7, 31–99)	n.s.
Anticoagulation	yes	13 (37.1%)	6 (37.5%)	n.s.
Type of anticoagulation				n.s.
	ASS	7 (20.0%)	3 (18.8%)	
	Clopidogrel	0 (0.0%)	0 (0.0%)	
	ASS+Clopid	1 (2.8%)	0 (0.0%)	
	VKA	2 (5.6%)	1 (6.7%)	
	NOAC	3 (8.5%)	2 (12.5%)	
History of previous stroke	yes	6 (17.1%)	2 (12.5%)	n.s.
History of AF^a^	yes	7 (20.0%)	3 (18.8%)	n.s.
CCI^b^				n.s.
	0–1	18 (51.4%)	9 (60.0%)	
	2–3	15 (42.8%)	6 (37.5%)	
	>3	2 (5.7%)	1 (6.7%)	
Art.hypertension	yes	17 (48.5%)	10 (62.5%)	n.s.
Diabetes	yes	5 (14.2%)	3 (18.8%)	n.s.
Renal disease	yes	1 (2.8%)	1 (6.7%)	n.s.
Liver disease	yes	2 (5.7%)	2 (12.5%)	n.s.
Pulmonary disease	yes	4 (11.4%)	2 (12.5%)	n.s.
Myocardial infarct /heart failure	yes	8 (22.8%)	3 (18.8%)	n.s.
PVD^c^	yes	3 (8.5%)	1 (6.7%)	n.s.
Cancer^d^	yes	5 (14.2%)	2 (12.5%)	n.s.

^a^*AF* Atrial fibrillation, ^b^*CCI* Charlson Comorbidity Index, ^c^PVD Peripheral vascular disease or bypass, ^d^ Non active cancer disease diagnosed at least 5 years before MT

The primary endpoint of our study was to investigate statistical differences for parameters of immunothrombosis, i.e. neutrophil markers, DNase-I and cytokines between stroke patients and healthy controls. After in vitro stimulation of patients’ and controls’ neutrophils we also aimed to reveal their different thrombogenic potential. A further clinical endpoint within the subgroup of stroke patients was short-term patients’ functional outcome assessed as NIHSS at discharge (favorable outcome defined as NIHSS 0–4). In addition, we investigated whether our studied laboratory parameters in serum, PBMC and thrombi were differentially expressed with distinct clinical characteristics or disparate applied therapy.

### Neutrophil isolation

Approx. 20 mL peripheral blood were obtained from stroke patients and healthy controls in blood collection tubes containing ethylenediaminetetraacetic acid (EDTA) and PBMCs as well as neutrophils were isolated by density gradient centrifugation (lymphocyte separation medium; 1077 GE Healthcare). Serum was collected in reaction tubes, PBMCs in 50 mL centrifuge tubes. Neutrophils were obtained by lysing erythrocytes according to previously published methods [19]. Based on this method, activation of neutrophils is minor and prevented by the use of ion-free buffers. Serum samples were stored at −80 °C until further use, whereas PBMCs were used directly for flow cytometric (FACS) analysis and purified neutrophils for FACS and cell culture experiments.

### Flow cytometry

Frequencies of LDG and monocytes within PBMCs as well as the cell number and purity of neutrophils were defined in single cell suspensions of 1x10^6^ cells/mL. Surface staining for anti-human: CD15-APC (BioLegend), CD14-PE (BioLegend), CD16-APC-Cy7 (BioLegend) and CD66b-FITC (BioLegend) was performed for 15 min at 4 °C. Cells were washed with phosphate buffered saline (PBS) and fixed with 4% paraformaldehyde (PFA). Viability dye (Zombie NIR, BioLegend) was used to determine dead cells.

Intracellular cytokine levels from CD4^+^ cells, were determined by stimulating PBMCs with 5 ng/mL phorbol 12-myristate 13-acetat (Sigma-Aldrich) and 500 ng/mL ionomycin (Sigma-Aldrich) for 5 h upon addition of Brefeldin A (BioLegend). Stimulation was stopped and cells were fixed with 4% PFA, treated with 0.05% saponin (Sigma-Aldrich) and stained intracellularly with anti-human: CD4-BV650 (BioLegend), IFN-y-PB (BioLegend), IL-17-PcP (BioLegend) and IL-22-PE-Cy7 (BioLegend) (all 1:100). Expression levels were determined via flow cytometry (BD FACS Canto II), obtained data analysed with FlowJo V10.1 and statistics calculated in GraphPad Prism 5.0.

### Stimulation of NET formation in vitro

NET formation was investigated *in vitro* by plating purified neutrophils at a density of 1x10^6^ cells/mL in RPMI Medium 1640 (Gibco, GE Healthcare) supplemented with 10 mM HEPES and 0.2% human serum albumin. Cells rested for 1 h at 37 °C and 5% CO_2_ in a poly-L-lysine (Sigma Aldrich) coated 24 well plate (Sarstedt GmbH). Afterwards neutrophils were treated with 100 mM PMA (positive control) or DMSO (negative control) for 3 h. Samples were subsequently either stained directly with 5 μM Sytox Green (Life Technologies) for 10 min and fixed with 4% PFA or treated with recombinant DNase-I (Roche) for 30 min before fixation. NET formation was assessed with fluorescence microscopy (AxioImager.Z2; Carl Zeiss) using 10x (numerical aperture 0.3) Plan-Neofluar magnification objectives. Images were analyzed with ImageJ software (version 1.52a).

### Quantification of NETosis with sytox green

Formation of NETs was quantified by plating 1x10^5^ neutrophils in 100 μL/well on a 96 flat-bottom well-plate in triplicates. Cells rested for 1 h at 37 °C and 5% CO_2._ Afterwards neutrophils were stimulated with 100 mM PMA (positive control), DMSO (negative control) or the different patients’ sera (50%) and incubated for 3 h. 5 μM Sytox Green (Life Technologies) was added to each well and incubated for 10 min. Fluorescence intensity was measured directly at a wavelength of 485/583 nm with a TECAN Infinite M1000 Pro plate reader.

### Determination of DNase-I

Quantification of DNase-I in sera of patients with ischemic stroke in comparison to the assessed controls was obtained by performing an ELISA for DNase-I (MBS285079; MyBioSource) according to the manufacturers protocol. Sera were diluted 1:3 during the density centrifugation gradient.

### Cryostat sectioning

Retrieved thrombi of patients with ischemic stroke were directly embedded in Tissue Teck O.C.T. Compound (Sakura Finetek) and stored at −80 °C until further processing. For cryostat sectioning samples were warmed up to −20 °C and two to three sections of 9 μm thickness were placed on each microscope slide. Up to 6 microscope slides were prepared and stored at −80 °C until immunofluorescence staining was performed.

### Immunofluorescence staining

Cryosamples were thawed, unspecific proteins were blocked with 5% Bovine Serum Albumin (BSA, Miltenyi Biotec) for 30 min, washed with PBS and stained with anti-human CD66b-PE antibody (BioLegend) and Sytox Green (Thermo Fisher Scientific) or DAPI (Thermo Fisher Scientific) and a rabbit-anti-human Cit-3H antibody (abcam) over night at 4 °C. Cit-H3 expression was visualized with a secondary goat-anti-rabbit-FITC antibody. IgG-PE and IgG-FITC (BioLegend) staining served as negative controls. Slides were mounted with antifade mounting medium (vectashield). Samples were examined on an AxioImager.Z2 (Carl Zeiss). Images were analyzed with ImageJ software (version 1.52a).

### Statistical analysis

The statistical analysis of our clinical and molecular data was performed using SPSS (version 26, IBM Deutschland, Ehningen, Germany) and GraphPad (Software Prism 5.0, San Diego, California, USA). Standard procedures were employed for univariate (Fisher exact test, Chi-square test, Shapiro-Wilk test, Kruskal-Wallis test, statistical significance was set to *p* < 0.05) and multivariate analyses (ANOVA with Bonferroni’s multiple comparison test, binary logistic regression, backward Wald stepwise method) as indicated (binary variables: NIHSS 0–4 vs. > 4; NIHSS improvement after MT yes/no). Continuous variables were presented as mean ± standard deviation (SD) or as median ± (interquartile range [IQR]) in case of non-normal distribution and were compared using the ANOVA or Mann Whitney U test, respectively. Categorical variables were analyzed using the Chi-square test. Correlation between inflammatory parameters was performed by Spearman Rho. All confidence intervals (CI) were assessed at 95.0%.

## Results

### Patient demographics

We reviewed 35 patients (18 males) with unilateral large artery occlusion. Median age was 77 years. In 14 patients (40.0%) the time of symptoms onset was longer than 4.5 h; wake up strokes have been noted in 6 patients (17.1%) (Table 1). 9 patients (25.7%) presented at admission with symptoms of minor (NIHSS 0–4), whereas 11 (31.4%) with symptoms of severe (NIHSS>16) stroke. (Table 2) 18 patients (51.4%) presented with a Charlson Comorbidity Index (CCI) of 0 or 1. A history of AF was known in 7 (20.0%) of our patients; in another 8 (22.8%) cases AF was first diagnosed during their hospitalization. 14 patients (40.0%) have been administered IVT prior to MT. Successful recanalization, i.e. TICI 2b, 2c or 3 has been achieved in all but 2 patients (94.3%). Our controls were comparable to our stroke patients in terms of all demographical and clinical parameters studied. The demographics of our stroke patients and controls are demonstrated in Tables 1 and 2.

**Table 2:** NIHSS and location of arterial occlusion

NIHSS	at admission	at discharge
0–4	9 (25.7%)	17 (48.5%)
5–15	15 (42.9%)	6 (17.1%)
>16	11 (31.4%)	12 (34.2%)
Occlusion of		
MCA	22 (62.9%)	
ACI	7 (20%)	
Basil/Vertebr.	6 (17.1%)	

### Differences of immunological phenotype between stroke patients and controls

**Fig. 1:**
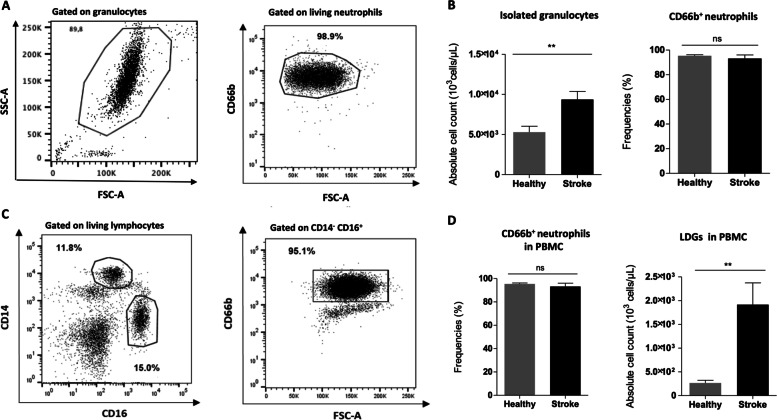
Stroke patients show increased numbers of neutrophils with an accumulation of Low Density Granulocytes. **A** Representative scatter plot of isolated neutrophils from a patient with an ischemic occlusion showing a mean purity of 98.9% with **B** equal frequencies of CD66b^+^ neutrophils in stroke patients (*n* = 35) compared to control patients (*n* = 16; Healthy). Total numbers of isolated granulocytes are significantly increased in patients with stroke when compared to their controls. **C** Representative scatter dot plots of flow cytometric assessed surface expression of CD14, CD16 and CD66b on PBMCs showing **D** significantly increased low density granulocytes (LDGs), defined as CD14^−^ CD16^+^ CD66b^+^ lymphocytes, in stroke patients, despite comparable frequencies of CD66b expression in both groups. Data are representative of independent experiments with 35 stroke patients and 16 control patients (Healthy) and presented as mean ± SD. Mann-Whitney-U Test, *p ≤ 0.05, **p ≤ 0.01, ***p ≤ 0.001. Abbreviations: LDG, low density granulocytes; PBMCs, peripheral blood mononuclear cells

**Fig. 2:**
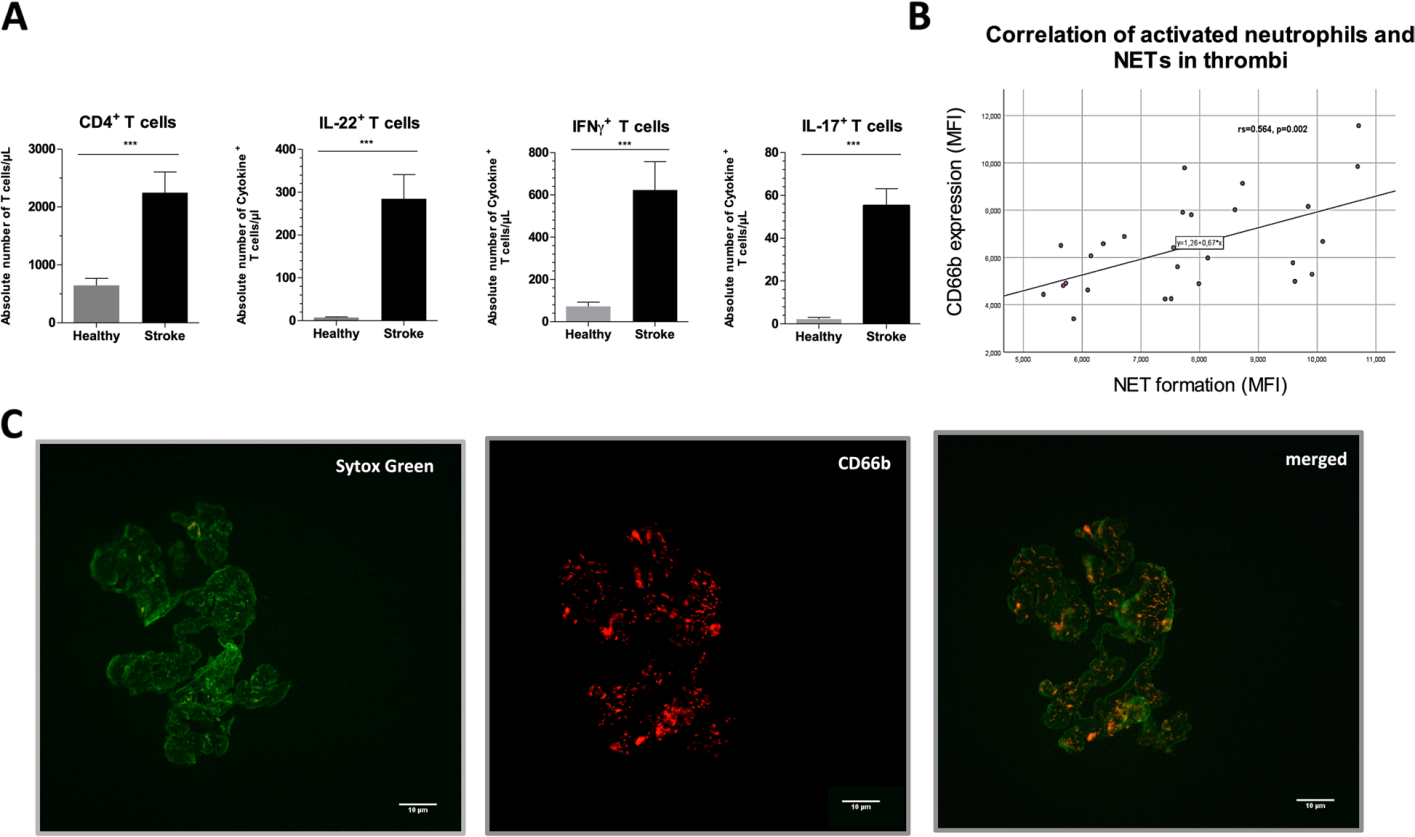
Stroke patients accumulate pro-inflammatory cytokines and show increased NET formation within the retrieved thrombi. **A** CD4^+^ T cells of patients with ischemic stroke and control patients were assessed for their intracellular expression of the pro-inflammatory cytokines IL-17, IFN-y and IL-22 and show significantly higher absolute numbers of these T_H_1 and T_H_17 cytokines in peripheral blood. Data are representative for 35 stroke patients and 16 control patients (Healthy) and presented as mean ± SD. Mann-Whitney-U Test, * *p* ≤ 0.05; ** *p* ≤ 0.01; *** *p* ≤ 0.001. **B** Retrieved thrombi of 35 ischemic patients were analyzed via fluorescence microscopy for the expression of intact but activated CD66b^+^ neutrophils within the thrombus and apoptotic neutrophils in form of NET (Sytox Green positive). The MFI for CD66b expression and NET formation within the thrombi were determined. Correlation between these two parameters shows a significant slope for such. **C** Representative fluorescence images of a thrombus stained for NET (green; left image) and CD66b (red; middle image) as well as an overly of both (merged; right image)

The comparison of the immunological values between stroke patients and healthy controls revealed statistically significant differences, i.e. stroke patients were more likely to express higher absolute counts of granulocytes (*p* = 0.007), DNAse-I (*p* < 0.001), LDG (*p* = 0.003) as well as increased CD4 counts (*p* = 0.005) and the CD4 related pro-inflammatory cytokines IL-17 (*p* < 0.001), INF-γ (*p* < 0.001) and IL-22 (*p* < 0.001) (Table 3) (Figs. 1, and 2). Cell counts of CD66b neutrophils were comparable between our two study groups (supplementary Fig. 1). After adjusting for all studied clinical characteristics, such as age, sex, type of anticoagulation, history of previous stroke or atrial fibrillation, the multivariate analysis recognized no independent prognosticator of stroke.

**Table 3:** Immunological profile in blood and PBMC of stroke patients and controls

	Stroke patients (*n* = 35)	Controls (*n* = 16)	
	Median (IQR)^b^	Median (IQR)	***U***^a^, *p* value
granulocytes 10^3^/μL	9.23 (5.88–20.73)	5.22 (3.97–7.33)	***128***, 0.007
CD66b + neutrophils 10^3^/μL	2.44 (21.75–30.25)	2.03 (10.67–28.80)	n.s
DNAse-I U/L	17.03 (13.91–20.72)	12.24 (10.59–13.20)	***24.5***, <0.001
IL-17-producing CD4^+^ cells/μl	64.9 (26.6–397.0)	3.18 (2.40–13.70)	***17***, <0.001
IL-22-producing CD4^+^ cells/μl	265.1 (132.1–775.1)	6.10 (3.81–9.53)	***25.5***, <0.001
INF-γ-producing CD4^+^ cells/μl	632 (172.75–1242,62)	54.24 (30.88–98.30)	***55***, <0.001
CD4 cells/μl	2467 (841–4678)	712 (420–991)	***94***, 0.005
LDG 10^3^/μL	1.93 (0.40–6.84)	0.24 (0.14–0.72)	***116***, 0.003

^a^Mann Whitney test *U*, ^b^non normal distribution

### Correlation of values of inflammatory parameters in serum and in thrombus

Higher relative proportions of CD66b in PBMC correlated with higher frequencies of NETs in thrombi of stroke patients (*r*_s_ = 0.472, *p* = 0.013). To this end, also the values of CD66b expressed in thrombi of stroke patients correlated with the counts of NETs in thrombi (r_s_ = 0.564, *p* = 0.002) (Fig. 2B).

### Association of clinical characteristics and immunological profile

Patients with onset time of symptoms longer than 4.5 h showed increased values of DNase-I (*U* = 42, *p* = 0.045) in sera as well as increased NET formation in thrombi (*U* = 30, *p* = 0.032). All studied comorbidities, intake of anticoagulants/antiplatelets, sex and age showed no influence upon values of immunothrombosis at admission.

### Association of immunological profile and NIHSS

Patients with NIHSS <5 at admission showed significant lower counts of neutrophils than stroke patients with higher NIHSS (*p* = 0.046). Lower values of IL-22 (*p* = 0.012) and monocytes (*p* = 0.039) in peripheral blood were observed in patients with improvement of NIHSS after MT. In addition, patients with lower CD66b in thrombi were more likely to benefit from the MT, as they showed a significant reduction of NIHSS post-intervention (*p* = 0.038). After adjustment for potential confounders, i.e. for sex, age, type of therapy (MT alone vs. MT with IVT) as well as for clinical (such as CCI, intake of anticoagulants/antiplatelets) and immunological (those with *p* < 0.05 in the univariate analysis) parameters, lower values of CD66b in thrombi were independently associated with greater improvement of NIHSS after MT (HR = 0.478 (0.232–0.985), *p* = 0.045 (logistic regression, backward Wald stepwise method).

A similar multivariate analysis (logistic regression, backward Wald stepwise) on potential prognostic factors for a more favorable short-term outcome, defined as NIHSS <5 at discharge, failed to recognize independent prognosticators.

### NET formation potential and thrombus composition

**Fig. 3:**
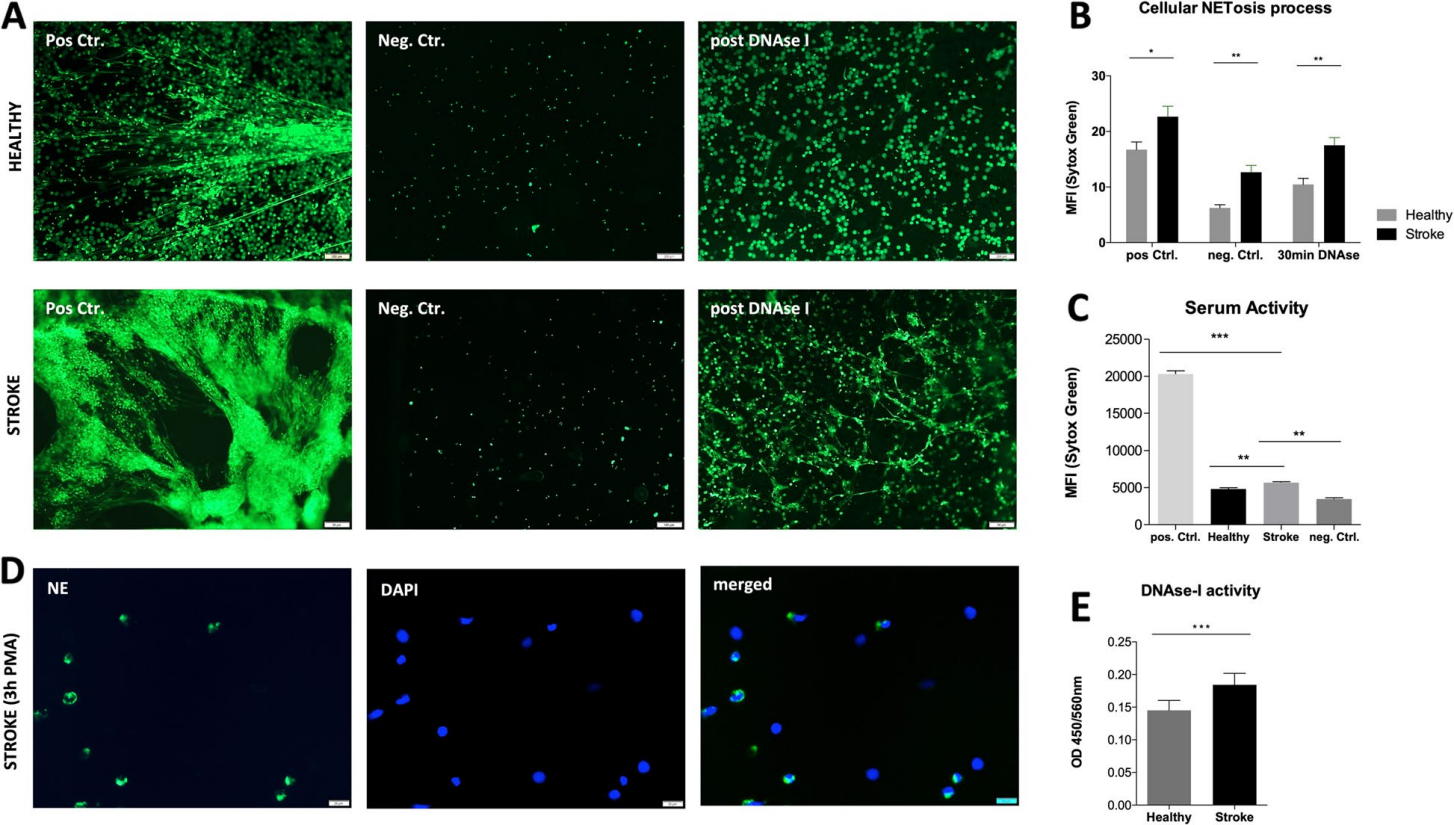
Neutrophils of stroke patients show elevated NET formation with worse resolution of already formed NET. **A** Representative images of neutrophils with or without NET formation for control patients (Healthy; upper row; *n* = 16) and stroke patients (Stroke; lower row; *n* = 35) Neutrophils are isolated from peripheral blood via ficoll density gradient and plated. NET formation is triggered by adding of 100 mM PMA (positive control) or DMSO (negative control) for 3 h. For the DNase-I control, samples are treated with 100 mM PMA for 3 h and afterwards with DNase-I for 30 min. Sytox Green is added to all samples 10 min before fixation with 4% PFA. NET formation is determined directly after fixation. **B** Quantification of the MFI (mean fluorescence intensity) of the Sytox Green positive images show significantly higher NET formation for patients with ischemic stroke for the positive control, but also already in resting neutrophils (negative control) and a worse resolution of NET upon DNAse-I treatment. **C** Neutrophils treated with serum of patients with ischemic stroke (3rd bar; *n* = 35) as well as serum of controls (2nd bar; *n* = 16) show significantly higher NET formation determined via MFI in comparison to the positive control (100 ng PMA) and the negative control (DMSO). **D** Represantative images of PMA stimulated neutrophils, showing nuclear expression (DAPI) of neutrophil elastase (NE) in green (anti-NE FITC) upon NETosis. **E** Serum of patients with ischemic stroke (3rd bar; *n* = 35) as well as serum of controls (2nd bar; *n* = 16) is analyzed for DNase-I levels and reveals higher levels of the enzyme in sera derived from stroke patients (*p* < 0.001). Data are representative of independent experiments with 35 stroke patients and 16 control patients (Healthy) and presented as mean ± SEM. Abbreviations: NET, neutrophil extracellular traps; MFI, mean fluorescence intensity. Mann-Whitney-U Test, * *p* ≤ 0.05; ** *p* ≤ 0.01; *** *p* ≤ 0.001

Neutrophils from patients with an ischemic occlusion as well as control patients could be isolated with a mean purity of 98.9%. Neutrophils from stroke patients showed significantly higher NET formation determined via MFI in comparison to control patients (Fig. 3A,B). The stimulation of neutrophils with PMA successfully induced neutrophil elastase (NE) activity, therefore a true NETosis (Fig. 3D). Further, sera obtained from ischemic stroke patients demonstrated a significantly higher potential to induce NET in untreated neutrophils despite significantly higher levels of DNase-I and worse clearance of already formed NET (Fig. 3C,E). The retrieved thrombi of the same patients showed an infiltration of CD66b^+^ neutrophils, which indicates the presence of still intact but activated cells about to promote NETosis. Further, NETosis is accompanied with Cit-H3 expression, which reflects a true NETosis process rather than just necrotic cells (supplementary Fig. 2A,B). However, within the same thrombi these neutrophils also presented a clear formation of NET mostly at the borders of the thrombus, but also initiating from the neutrophil-accumulations and the center of the thrombus.

### Association of IVT and immunological profile

Stroke patients applied MT and IVT vs. MT alone showed a trend for lower values of NET in thrombi (*p* = 0.078) as well as higher counts of DNAse-I in serum (*p* = 0.004). After a subgroup comparison of either 21 stroke patients administered IVT vs. 16 controls or 14 stroke patients without IVT vs. 16 controls the above documented differences (please see ‘*Differences of immunological phenotype between stroke patients and controls’)* remained still significant.

## Discussion

A potential involvement of neuroinflammation in stroke has long been conceived. Increased WBC counts after stroke have been a common notion [20]; moreover, leukocyte counts were reported to be inverse associated with the clinical outcome in acute ischemic stroke patients [21]. Apart from the abnormalities of the obviously main player of the immunological response, i.e. leukocytes [22], further systemic changes in specific inflammatory parameters, such as cytokines and chemokines have been reported [23–26]. Brain ischemia, cellular death and oxidative stress after ischemic stroke are believed to initiate an intense inflammatory response resulting in a transient immunosuppression [26–29].

However, it is a legitimate question whether the observed neuroinflammation reflects only the response to an established stroke or may also contribute to its genesis, i.e. whether neuroinflammation is also the cause and not only the consequence of stroke [8–10]? Novel insights point towards a pivotal role of inflammation, in particular of NET, in generating venous and arterial thrombosis [30, 31]. Dyer et al. reported that NET formation is a primary regulator of deep vein thrombosis in mice [32]. Release of NET has been evolved also in the genesis of arterial thrombosis of both coronary as well as intracranial arteries [6, 8, 9, 11–13, 33]. Genchi et al. found increased content of NET in cerebral thrombi of cardioembolic etiology [34]. Klopf et al. support that the cascade of NETosis in stroke may be triggered by alterations of granulopoiesis and subsequently induction of immature LDG, which then undergo spontaneous NET formation [35].

This new knowledge may expand our understanding concerning thrombogenesis in stroke patients. The so called TOAST classification categorizes potential sources of thrombosis; apart from the cardioembolic and atherosclerotic causes, a wide variety of stroke cases remains debatable [36]. According to the clinical experience 25–39% of strokes remain without certain cause [37]. The lack of comprehensive understanding of stroke pathophysiology is being reflected by the insufficient pharmacological therapy, to date. In cases with large artery strokes less than half of the patients respond to IVT. The identification of immunothrombosis as possible cause of stroke and the encryption of the mechanisms of immune related thrombogenesis may allow a more targeted stroke therapy. Mangold et al. showed a positive association of coronary NET burden with myocardiac infarct size and an accelerated lysis of coronary thrombi *ex vivo* after addition of DNAse-I [33]. Laridan et al. as well as Ducroux et al. detected NET in ischemic stroke thrombi and reported a more powerful thrombolysis *ex vivo* after combined rtPA and DNAse-I administration than rtPA alone [9, 38]. Thus, above studies identify NET as potential pharmacological target in order to optimize the IVT efficiency.

Our study though aimed to analyze the role of NET, i.e. the efficacy of DNAse-I on NET clearance and the pathogenesis of NET release from another aspect. Since NET formation in thrombi reflects a systemic alteration of the immune system, the formation and the degradation of NETs in stroke patients may be abnormal also in the periphery. To this end, we compared the potential of neutrophils of stroke patients vs. healthy controls to promote NET formation and clearance of already formed NET after DNase-I application *in vitro* and found prominent differences. Neutrophils of stroke patients showed indeed a higher formation of NET and a worse resolution upon DNAse-I treatment when NETs were already formed compared to neutrophils derived by healthy individuals (Fig. 3). This observation may support the hypothesis that neutrophil dysfunctions may be apparent before the event of stroke and NET formation may contribute to the initial thrombogenic event of a stroke rather than just being released as part of the immune response. Under this aspect, NET may be identified as potential therapeutic target not only during the IVT but more importantly, in terms of prophylaxis. The latter should preferentially aim towards selective targets, like PAD4, which reflects an essential factor for NET formation and not towards general immune pathways, since immunosuppressive drugs may increase the risk of bacterial or viral infections. Even in such cases, patients under PAD4 blockage medication should be carefully selected and closely monitored.

Further indices for a potential involvement of NET in the genesis of ischemic stroke thrombi is given by the fact that in our series patients with longer onset time of symptoms showed higher values of NET in their thrombi but also higher values of DNase-I in their sera, a known degrading enzyme of NET [9, 39, 40], probably as an intrinsic systemic lytic reaction against formation of thrombus. Noteworthy, the values of DNase-I in the sera seem not to be constant but highly dependent on the state of the current immune response. In our series patients with already formed NET showed higher values of DNase-I in the acute phase of stroke. However, Kim et al. studied chronic hemodialysis patients and found no correlation between values of NET and DNase-I in their serum, whereas Vaibhav et al. reported reduced DNase-I activity in patients after traumatic brain injury [41, 42].

As shown before, parameters of neuroinflammation are abnormally expressed in patients with stroke [23, 25, 43, 44]. We conducted a case - control study, to investigate for differences of their immunological profile between stroke and control patients admitted at hospitals for degenerative spinal pathologies. Our study revealed significant differences, i.e. higher absolute counts of neutrophils, LDGs, as well as IL-22, IL-17 and IFN-γ producing CD4^+^ T cells favoring a T_H_1/T_H_17 response and pro-inflammatory environment. Such high levels of cytokines and other yet unidentified molecules can affect neutrophil development in the bone marrow and contribute to a quick mobilization of neutrophils thereby generating rather LDG than normal density granulocytes (NDG), going in line with our results showing increased LDG levels in stroke patients (Fig. 1). LDG have already been described with an enhanced capacity to form spontaneous NETs [45]. Systemic and local secretion of pro-inflammatory cytokines has been reported earlier [46, 47]. Our results confirm previous reports of increased values of pro-inflammatory cytokines in the acute phase of ischemic stroke.

After performing Spearman rho statistics to conclude for correlations between the immune values in blood and thrombi of the assessed patients we observed a significant correlation of CD66b in both, blood and thrombi with counts of NETs in thrombi. As CD66b constitutes a surface marker for neutrophil activation, we speculate that immunothrombosis may be promoted by an increased the NETosis and subsequent formation of thrombi by abnormally activated neutrophils. Under normal circumstances, neutrophils patrol throughout their short lifespan through the arteries without adhering to the vascular endothelium to migrate towards tissues. However, certain neutrophil surface markers, such as CD66b, can influence the adhesion of this cells to the endothelium and activate them leading to subsequent apoptotic NET formation [48, 49].

The associations found between parameters of immunothrombosis and the response of patients to therapy are of great interest. Novotny et al. analyzed 71 thrombi retrieved from patients with acute ischemic stroke and found poor outcome scores in patients with abundant amounts of NET [50]. Ilie et al. reported a prognostic value of CD66b, since infiltrating CD66b neutrophils in solid tumors [51] showed inverse association with patients’ outcome. In principle, higher CD66b or NET values may represent a malign stroke parameter, as increased levels may contribute to greater thrombus formation and may be related to worse outcome. To this end, we documented a clear positive correlation of CD66b and NET values in our studied thrombi. Of note, in our series patients with lower values of CD66b in thrombi and in peripheral blood are more possible to experience a significant reduction of NIHSS, thus a better neurological outcome, after MT. This observation is in line with the hypothesis supported before that firmer thrombus composition, in particular NET dominant thrombi may show higher resistance to mechanical removal [52, 53].

We have to acknowledge that our study is subject to several limitations, such as the sample size, which does not allow far reaching conclusions concerning the alterations of the immunity in stroke patients. In addition, our study does not provide information about long term patients’ outcome. However, our project shows several strengths, such as its prospective design and the balanced case-control study. Further, we analyzed not only the expression, but also the function of specific parameters of immunothrombosis in peripheral blood and thrombi.

## Conclusions

This study provides new insights into the role of activated neutrophils, LDG and NET formation in ischemic stroke. The observed impairments of the immune system may be apparent also prior to an event of stroke and predispose to the thrombogenic occlusion of large intracranial arteries. Thus, NET and CD66b may be identified as potential pharmacological targets in the therapy of stroke. Nevertheless, larger prospective studies are required to decipher the role of immunothrombosis in the genesis of stroke.

## Supplementary Information

**Additional file 1.**

**Additional file 2.**

## Data Availability

The datasets used and/or analyzed during the current study are available from the corresponding author on reasonable request.
