# Correction to: Stroke‑derived neutrophils demonstrate higher formation potential and impaired resolution of CD66b + driven neutrophil extracellular traps

**DOI:** 10.1186/s12883-023-03285-5

**Published:** 2023-06-23

**Authors:** Angeliki Datsi, Laura Piotrowski, Markella Markou, Thomas Köster, Isabelle Kohtz, Kerstin Lang, Sabine Plöttner, Heiko Udo Käfferlein, Burkhard Pleger, Ramon Martinez, Bogdan Pintea, Roland Fried, Marcus Müller, Rene Chapot, Konstantinos Gousias

**Affiliations:** 1 Institute for Transplantation Diagnostics and Cell Therapeutics, Heinrich-Heine-University Düsseldorf, 40225 Düsseldorf, Germany; 2 Medical School, Rheinische Friedrich-Wilhelms University of Bonn, Sigmund Freud Strasse 25, 53121 Bonn, Germany; 3 Department of Neurology and Psychotraumatology, BG Klinikum Duisburg, Großenbaum Allee 250, 47249 Duisburg, Germany; 4 Department for Diagnostic and Interventional Radiology, University Hospital Bonn Venusberg-Campus 1, 53127 Bonn, Germany; 5 Ruhr University Bochum, Universitätsstraße 150, Bergmannsheil Bochum, 44801 Bochum, Germany; 6 Institute for Prevention and Occupational Medicine (IPA) Ruhr University Bochum (IPA), Bochum, Germany; 7 Department of Neurology, University Hospital Bergmannsheil Bochum, Bürkle‑de‑la Camp Platz 1, 44079 Bochum, Germany; 8 Department of BG Neurosurgery and Spinal Surgery, University Hospital Bergmannsheil Bochum, Bürkle‑de‑la Camp Platz 1, 44079 Bochum, Germany; 9 Statistics in the Biosciences, TU Dortmund University, Vogelpothsweg 87, 44221 Dortmund, Germany; 10 Department of Neurology, St Marien Academic Hospital Hamm, St Paulus Corporation, Knappenstrasse 19, 59071 Hamm, Germany; 11 Department of Radiology and Neuroradiology, Alfried‑Krupp‑Hospital Rüttenscheid, 45131 Essen, Germany; 12 Department of Neurosurgery, KLW St Paulus Corporation, St Marien Academic Hospital Lünen, Westfälische Wilhelms-University Münster, Altstadtstrasse 23, 44534 Lunen, Germany; 13 Medical School, University of Münster, Domagkstrasse 3, 48149 Münster, Germany; 14 Medical School, University of Nicosia, Ilia Papakyriakou 21, 2414 Nicosia, Cyprus

**Correction: BMC Neurol 22, 186 (2022)**

10.1186/s12883-022-02707-0

Following publication of the original article [1], an error was identified in the Authors’ contributions section.

The updated conclusion is given below and the changes have been highlighted in bold.

**Authors’ contributions**

AD, **LP**, RC and KG designed and conceptualized the study; AD, LP, MM, TK, IK, BP and KG acquired data; AD, LP, MM, TK, IK, BP, RF, RC and KG analyzed data; RF, **LP and KG** conducted the statistical analysis, AD, **LP** and KG drafted the manuscript for intellectual content; AD, LP, MM, TK, IK, KL, SP, HUK, BP, RM, BP, RF, MM, RC and KG revised the manuscript for intellectual content. The author(s) read and approved the final manuscript.

The original article [1] has been updated.
